# Targeting Mutant Kirsten Rat Sarcoma Viral Oncogene Homolog in Non-Small Cell Lung Cancer: Current Difficulties, Integrative Treatments and Future Perspectives

**DOI:** 10.3389/fphar.2022.875330

**Published:** 2022-04-20

**Authors:** Jia-Xin Li, Run-Ze Li, Lin-Rui Ma, Peng Wang, Dong-Han Xu, Jie Huang, Li-Qi Li, Ling Tang, Ying Xie, Elaine Lai-Han Leung, Pei-Yu Yan

**Affiliations:** ^1^ State Key Laboratory of Quality Research in Chinese Medicines, Faculty of Chinese Medicine, Macau University of Science and Technology, Macao, China; ^2^ State Key Laboratory of Dampness Syndrome of Chinese Medicine, The Second Affiliated Hospital of Guangzhou University of Chinese Medicine (Guangdong Provincial Hospital of Chinese Medicine), Guangdong Provincial Academy of Chinese Medical Sciences, Guangzhou, China; ^3^ School of Traditional Chinese Medicine, Southern Medical University, Guangzhou, China; ^4^ Guangdong Provincial Key Laboratory of Chinese Medicine Pharmaceutics, Guangzhou, China; ^5^ Guangdong Provincial Engineering Laboratory of Chinese Medicine Preparation Technology, Guangzhou, China; ^6^ Zhuhai Hospital of Integrated Traditional Chinese and Western Medicine, Zhuhai, China; ^7^ Dr. Neher’s Biophysics Laboratory for Innovative Drug Discovery, Macau University of Science and Technology, Macao, China

**Keywords:** non-small cell lung cancer, KRAS, covalent KRAS^G12C^ inhibitor, immunotherapy, natural compound, combination treatment

## Abstract

In the past few decades, several gene mutations, including the anaplastic lymphoma kinase, epidermal growth factor receptor, ROS proto-oncogene 1 and rat sarcoma viral oncogene homolog (RAS), have been discovered in non-small cell lung cancer (NSCLC). Kirsten rat sarcoma viral oncogene homolog (KRAS) is the isoform most frequently altered in RAS-mutated NSCLC cases. Due to the structural and biochemical characteristics of the KRAS protein, effective approaches to treating KRAS-mutant NSCLC still remain elusive. Extensive recent research on KRAS-mutant inhibitors has made a breakthrough in identifying the covalent KRAS^G12C^ inhibitor as an effective agent for the treatment of NSCLC. This review mainly concentrated on introducing new covalent KRAS^G12C^ inhibitors like sotorasib (AMG 510) and adagrasib (MRTX 849); summarizing inhibitors targeting the KRAS-related upstream and downstream effectors in RAF/MEK/ERK pathway and PI3K/AKT/mTOR pathway; exploring the efficacy of immunotherapy and certain emerging immune-related therapeutics such as adoptive cell therapy and cancer vaccines. These inhibitors are being investigated in clinical trials and have exhibited promising effects. On the other hand, naturally extracted compounds, which have exhibited safe and effective properties in treating KRAS-mutant NSCLC through suppressing the MAPK and PI3K/AKT/mTOR signaling pathways, as well as through decreasing PD-L1 expression in preclinical studies, could be expected to enter into clinical studies. Finally, in order to confront the matter of drug resistance, the ongoing clinical trials in combination treatment strategies were summarized herein.

## Introduction

Cancer arises as a result of the accumulation of mutations in cancer-related genes, known as cancer drivers. In the last few decades, several types of genetic mutations have been observed in non-small cell lung cancer (NSCLC), including genomic alterations in anaplastic lymphoma kinase (ALK), epidermal growth factor receptor (EGFR), ROS proto-oncogene 1 (ROS1) and rat sarcoma viral oncogene homolog (RAS) (Lee et al., 2016). The RAS family is a commonly-mutated oncogene family in NSCLC, accounting for >30% of all oncogenes, causing ∼1 million deaths worldwide annually. More effective drugs are therefore needed to target the RAS mutation. However, due to its special structure, nearly no drug is perceived to be effective against the RAS mutation (Cox et al., 2014).

**FIGURE 1:**
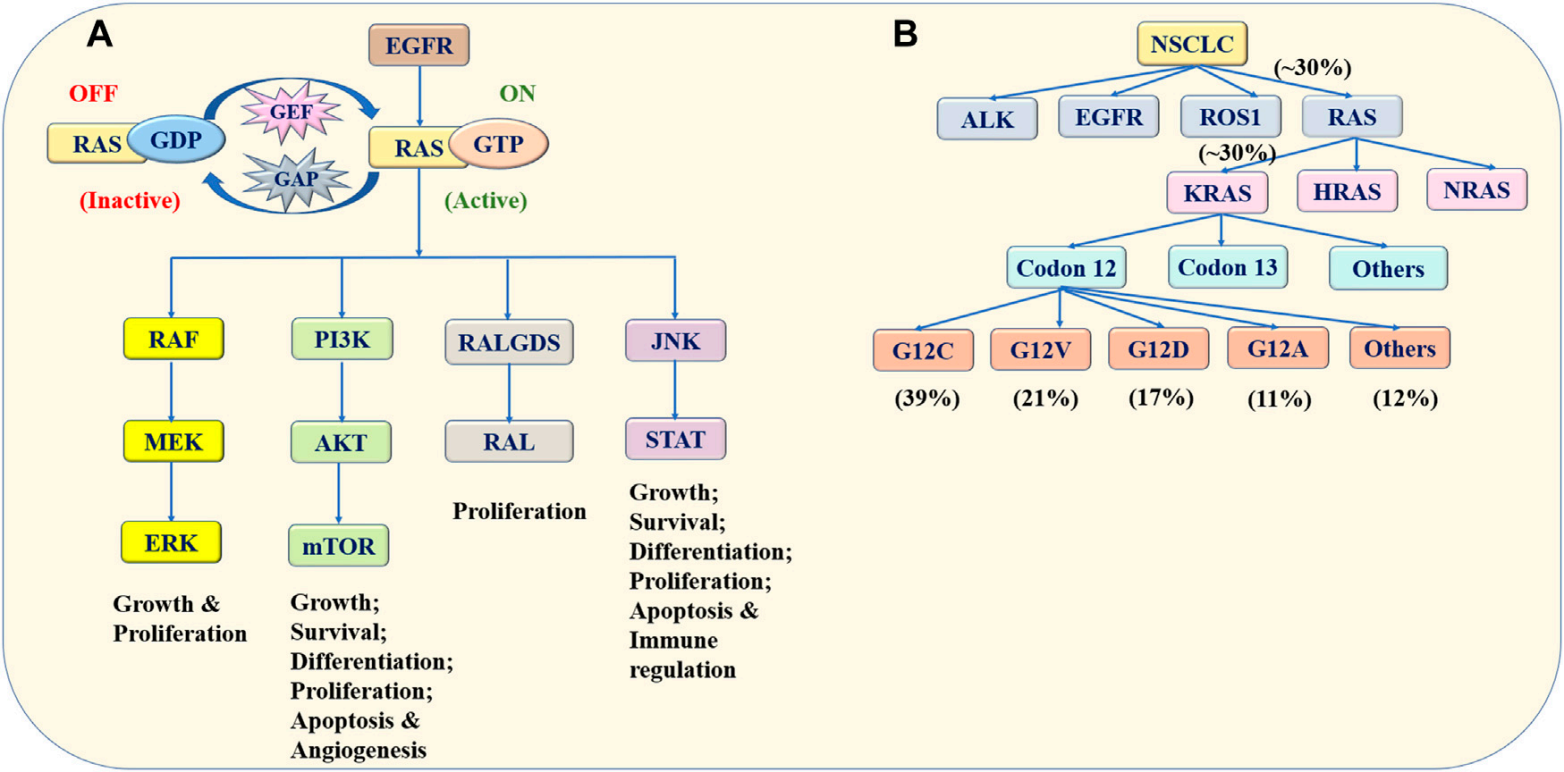
Schematic presentation of the RAS family. **(A)** Switching mechanism and signaling pathways of the RAS protein. The RAS protein switches between the GDP-binding inactive state and GTP-binding active state influenced by GAP and GEF. Once stimulated by external signals, such as the EGFR mutation, RAS/GDP changes into RAS/GTP, leading to the hyperactivation of downstream signaling cascades. The downstream pathways of the RAS protein include the RAF/MEK/ERK, PI3K/AKT/mTOR, RALGDS/RAL and JNK/STAT pathways, which are associated with tumor cell proliferation, differentiation, apoptosis and survival. **(B)** Frequency and classification of RAS mutation in NSCLC. KRAS, HRAS and NRAS are members of the RAS family, which is associated with NSCLC. Among them, the KRAS mutation is the most common mutation. The KRAS mutation exists mainly at codon 12. The G12C, G12V, G12D, and G12A mutations account for 39, 21, 17, and 11% of all KRAS mutations, respectively. Data acquired from Memorial Sloan-Kettering Cancer Center. Abbreviation: RAS, rat sarcoma viral oncogene homolog; GDP, guanosine diphosphate; GTP, guanosine triphosphate; EGFR, epidermal growth factor receptor; RAF, rapidly accelerated fibrosarcoma; MEK, extracellular regulated protein kinase; ERK, extracellular regulated protein kinase; PI3K, phosphatidylinositol 3 kinase; mTOR, mammalian target of rapamycin; RALGDS, Ral Guanine Nucleotide Dissociation Stimulator; RAL, Ras-related GTPase; JNK, c-Jun N-terminal kinase; STAT, signal transducer and activator of transcription; NSCLC, non-small cell lung cancer; KRAS, Kirsten rat sarcoma viral oncogene homolog; HRAS, Harvey rat sarcoma viral oncogene; NRAS, Neuroblastoma rat sarcoma viral oncogene.

The RAS protein family switches between the guanosine diphosphate (GDP)-binding inactive state and the guanosine triphosphate (GTP)-binding active state (Kranenburg, 2005). Under normal physiological conditions, the RAS protein is usually present in the form of GDP binding. Once stimulated by external signals, RAS-GDP changes into RAS-GTP. This leads to the hyperactivation of downstream signaling cascades, resulting in the uncontrolled proliferation and survival of tumor cells (Cox and Der, 2010). The EGFR family is the upstream effector of RAS, and therefore the mutation of this receptor tyrosine kinase promotes RAS activation. The downstream pathways of the RAS protein mainly include RAF/MEK/ERK pathway and PI3K/AKT/mTOR pathway. The RAS protein is also related to RALGDS-RAL pathway and JNK-STAT pathway. These pathways are associated with cell proliferation, anti-apoptosis, differentiation and survival (Hofer et al., 1994; Khosravi-Far et al., 1995; Gupta et al., 2007; Uras et al., 2020; Niloy et al., 2021) (Figure 1A).

Three genes, including Kirsten rat sarcoma viral oncogene (KRAS), Harvey rat sarcoma viral oncogene (HRAS) along with Neuroblastoma rat sarcoma viral oncogene (NRAS), are members of the RAS family, which are associated with cancers. Among them, KRAS is the most common mutation, which has been observed in ∼30% of patients with NSCLC (Skoulidis and Heymach, 2019). Furthermore, the KRAS mutation exists mainly at codons 12 and 13. A large research conducted in Memorial Sloan-Kettering Cancer Center found that the most ordinary codon variant was G12C mutation, accounting for 39% of KRAS mutations. G12V, G12D, and G12A mutations accounted for 21, 17, and 11% of KRAS mutations, respectively (Dogan et al., 2012) (Figure 1B).

Currently, identifying effective therapies to target the KRAS-mutant NSCLC is challenging, due to the structural and biochemical properties of the KRAS protein. Several possible factors are listed below. First of all, the surface of the KRAS protein is too smooth to bind with other small molecule inhibitors. Secondly, the high-affinity binding of GTP to the KRAS protein is difficult to break (Dang et al., 2017). In addition, there is little structural difference between wild type and mutant KRAS. So, drugs which target KRAS mutation usually affect normal KRAS (Nagasaka et al., 2020). The above obstacles make the direct targeting of KRAS challenging. On the other side, using small molecules to indirectly target the upstream and downstream effectors of the KRAS pathway remains a difficult task. KRAS-related pathways are complex and with several regulatory feedback loops. Mutated KRAS proteins can bypass specific molecules, resulting in a low or no drug-induced inhibition of KRAS signaling (Spiegel et al., 2014). Research is ongoing on ways to overcome these difficulties. The aim of the present review is to summarize the current progress of various treatments against KRAS-mutated NSCLC. First, several covalent KRAS^G12C^ inhibitors are described, especially sotorasib (AMG 510) and adagrasib (MRTX 849). Next, inhibitors targeting the KRAS-related upstream and downstream effectors are summarized, including the most common RAF/MEK/ERK pathway and PI3K/AKT/mTOR pathway. Next, immunotherapy along with other emerging therapies, such as adoptive cell therapy and cancer vaccines, are listed. At the same time, the use of compounds extracted from natural plants and herbs is also a potential option, due to their safety and multi-targeted properties. Finally, some combination treatment strategies are currently under evaluation to determine their efficacy and safety, in order to make sure an effective treatment for KRAS mutation.

## Direct and Indirect Attempts to Target Kirsten Rat Sarcoma Viral Oncogene Homolog in Treating Non-Small Cell Lung Cancer

KRAS has historically been regarded as an undruggable target. Fortunately, with the persistent efforts of researchers, new inhibitors directly or indirectly targeting KRAS have been clinically tested. This section mainly focuses on covalent KRAS^G12C^ inhibitors and inhibitors targeting the main KRAS-related pathways.

### Studies on Covalent Kirsten Rat Sarcoma Viral Oncogene Homolog^G12C^ Inhibitors

KRAS^G12C^ is the most common mutation in KRAS-mutant NSCLC. Two covalent KRAS^G12C^ inhibitors, sotorasib and adagrasib, are currently highly anticipated.

Sotorasib was developed by Amgen. It received accelerated approval from the United States FDA on 28 May 2021 for treating patients with KRAS^G12C^-mutant NSCLC (Blair, 2021). This irreversible inhibitor locks KRAS^G12C^ in the inactive GDP-bound state and binds to a special groove created by histidine 95 (Gentile et al., 2017). The recommended dosage is 960 mg orally once a day and its half-life is 5.5 h (Nagasaka et al., 2020). In a multicenter CodeBreaK 100 clinical trial (NCT03600883), the results showed that among the 124 patients with NSCLC who had previously received chemotherapy and/or immunotherapy, the objective response rate (ORR) reached 36% and 81% of patients achieved tumor control. The median progression-free survival (PFS) was 6.8 months and the median overall survival (OS) was 12.5 months (Li B. et al., 2021). Thus, sotorasib successfully fills the gap in the clinical treatment of KRAS^G12C^ NSCLC. The most common adverse effects are diarrhea, musculoskeletal pain, nausea, fatigue, and cough (Li B. et al., 2021). In addition, some randomized, open-label, multicenter trials on sotorasib are underway or recruiting patients, including NCT04303780, NCT04933695, NCT04625647, NCT05054725, NCT04185883, NCT04380753, and NCT04667234 (Blair, 2021).

Adagrasib was developed by Mirati Therapeutics. It binds to cysteine 12 within the Switch II pocket and also irreversibly locks KRAS^G12C^ in the inactive GDP-bound state. The recommended dosage is 600 mg orally twice a day and its half-life is 24.7 h (Hellmann et al., 2019). In an ongoing, multicenter, phase I/II trial (NCT03785249), 3/4 responders had NSCLC. Following adagrasib treatment, no patient exhibited brain metastasis. Adagrasib had better safety and the most common adverse events are grade 1 or 2 diarrhea, and nausea (Janne et al., 2019). In addition, certain randomized, open-label, multicenter trials are underway or recruiting patients, including NCT04613596, NCT04685135, NCT04330664, and NCT04975256. The basic features of sotorasib and adagrasib are shown in Table 1. Due to the small number of participants in clinical trials, the advantages of these two inhibitors are not comparable at present.

**TABLE 1:** Comparison between AMG 510 and MRTX 849.

Compound	AMG 510	MRTX 849
Alternative name	Sotorasib; lumakras™	Adagrasib
Molecular formula	C_30_H_30_F_2_N_6_O_3_	C_32_H_35_ClFN_7_O_2_
Structure	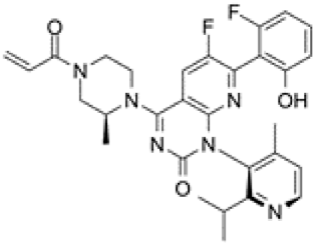	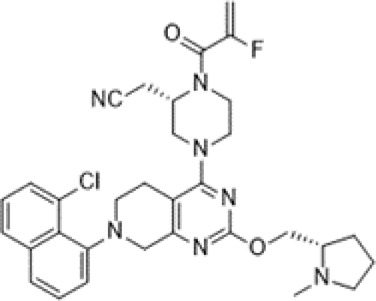
Key features	This irreversible inhibitor locks KRAS^G12C^ in an inactive GDP-bound situation and it can bind to a special groove created by His95	This small molecule inhibitor binds to Cysteine 12 in the Switch II pocket of KRAS^G12C^ and irreversibly locks KRAS^G12C^ in an inactive GDP-bound situation
Recommended dosage	960 mg once daily with oral	600 mg twice a day with oral
Half life	5.5 h	24.7 h
Adverse effect (AE)	Common AEs: diarrhea, musculoskeletal pain, nausea, fatigue	Common AEs: grade 1 or 2 diarrhea or nausea
	Grade 3 or 4 AEs: hepatotoxicity, increased ALT, increased AST and pneumonia	
Clinical trial identifier	NCT03600883, NCT04303780, NCT04933695, NCT04625647, NCT05054725, NCT04185883, NCT04380753, and NCT04667234	NCT03785249, NCT04613596, NCT04685135, NCT04330664, and NCT04975256

ALT, alanine aminotransferase; AST, aspartate aminotransferase.

In addition, a number of new KRAS^G12C^ inhibitors are under development. Patients with advanced KRAS ^G12C^-positive solid tumors were selected for the phase I trial for inhibitor JNJ-74699157 (NCT04006301). That trial was completed with promising results. Furthermore, LY3499446 and LY3537982, developed by Eli Lilly, have been included in NCT04165031 and NCT04956640, respectively (Nagasaka et al., 2020). Clinical trials on GDC-6036 and D-1553 are at the recruitment stage (NCT04449874 and NCT04585035) (Addeo et al., 2021).

However, acquired resistance to KRAS^G12C^ inhibitors still occurs. Resistance mechanisms have been previously described (Zhang et al., 2022). First and foremost, KRAS on-target mutations, including R68S, H95D/Q/R, G12D/R/V/W, G13D, Q61H and Y96C, disrupt the drug-binding interface (Awad et al., 2021). Next, KRAS amplification along with secondary mutations of KRAS^G12C^ (G12D/R/V/W) induce drug resistance (Skoulidis et al., 2021). Moreover, reactivating upstream and downstream pathways has been confirmed to be the reason for that drug resistance (Hellmann et al., 2019; Xue et al., 2020). Meanwhile, histological transformation, such as from adenocarcinoma to squamous cell carcinoma, is a non-genetic mechanism (Adachi et al., 2020). Finally, activating the dysregulation of cell cycle and focal adhesion kinase (FAK) signaling is a potential resistance mechanism (Tang et al., 2016; Hellmann et al., 2019). We therefore hope that an increasing number of promising drugs that can battle acquired resistance will be identified in the future.

### Studies on Inhibitors Targeting the Main Kirsten Rat Sarcoma Viral Oncogene Homolog-Related Pathways

The activation of KRAS upstream pathway signals may lead to the activation of KRAS mutations, which may cause continuous stimulation of its downstream signaling pathways and promote tumorigenesis (Patricelli et al., 2016). Thus, suppressing the upstream and downstream effectors of the KRAS pathways, including EGFR, rapidly accelerated fibrosarcoma (RAF), MAPK/ERK kinase (MEK), extracellular regulated protein kinase (ERK), phosphatidylinositol 3 kinase (PI3K), mammalian target of rapamycin (mTOR) and FAK, may be an effective therapeutic method. Certain single-agent inhibitors which have been studied in clinical trials are listed as follows.

#### Epidermal Growth Factor Receptor Inhibitors

EGFR is the upstream effector of KRAS. Certain EGFR tyrosine kinase inhibitors (TKIs), such as cetuximab are usually used to treat KRAS^G12D^-mutated metastatic colorectal cancer. Patients often have increased PFS and OS (De Roock et al., 2010). However, few EGFR-TKI is effective in treating KRAS-mutant NSCLC due to the special resistance (Pao et al., 2004; Kobayashi et al., 2005; Linardou et al., 2008; Mao et al., 2010). In the case reports written by Dr. Xiu’s team, KRAS^G12S^-mutant NSCLC patients treated with the third-generation EGFR-TKI osimertinib experienced rapid disease progression instead (Xiu et al., 2021). Fortunately, combination treatment with EGFR-TKI and other inhibitors has been found to effectively treat KRAS-mutant NSCLC, as shown in the section below.

#### Rapidly Accelerated Fibrosarcoma Inhibitors

Active GTP-bound KRAS mutation promotes the phosphorylation of RAF, thereby inducing the phosphorylation of the RAF substrate MEK. To effectively treat KRAS-mutant NSCLC, the RAF/MEK/ERK pathway needs to be inhibited. Sorafenib is a multi-targeted inhibitor. For example, in a phase I, open-label, single-center trial, 10 patients with various types of KRAS-mutant advanced NSCLC were tested. The results showed a PFS of ∼3 months (Smit et al., 2010). In a single-arm, phase II, multi-center trial, patients were administered 400 mg sorafenib twice daily. The PFS was 2.3 months along with OS was 5.3 months (Dingemans et al., 2013). In a BATTLE program, the median PFS was 2.83 months and median OS was 8.48 months (Blumenschein et al., 2013). Similarly, in another BATTLE trial, the median PFS was 1.9 months while the median OS was 8.8 months (Kim et al., 2011). In a phase III, placebo-controlled, multi-center trial, the PFS was longer in the KRAS-mutant group compared with the KRAS wild-type subpopulation (Paz-Ares et al., 2015). Furthermore, in a phase II, randomized BATTLE-2 trial, OS and PFS were similar for patients with a KRAS mutation or wild-type NSCLC (Papadimitrakopoulou et al., 2016). In short, common adverse events following sorafenib treatment mainly included hand-foot syndrome, diarrhea, fatigue, and rash. PFS was less than 3 months while OS was shorter than 9 months (Smit et al., 2010; Kim et al., 2011; Blumenschein et al., 2013; Dingemans et al., 2013; Paz-Ares et al., 2015; Papadimitrakopoulou et al., 2016). In addition to sorafenib, RO5126766 also targets RAF, which is a dual MEK/RAF inhibitor. For instance, the results of a phase I, multi-center, dose-escalation study showed that inhibition by oral RO5126766 was a feasible anti-NSCLC method, despite the appearance of adverse events such as rash, elevation of creatine phosphokinase, diarrhea and blurred vision (Martinez-Garcia et al., 2012). Meanwhile, in a phase I, single-center, non-randomized trial, the tolerability of RO5126766 in Japanese patients was 2.25 mg/day once daily, the same as in European patients. Adverse events were also similar. However, the half-life time was longer (Honda et al., 2013). In another phase I, open-label, single-center study, tumor regression was observed in 60% of patients, with two patients maintaining a response for >1 year, which suggested that the RO5126766 showed preclinical potential (Chenard-Poirier et al., 2017).

#### MAPK/Extracellular Regulated Protein Kinase Inhibitors

MEK is a serine/threonine kinase, activated by RAF. PD-0325901 is an oral, small molecule inhibitor targeting MEK. In a phase II, multi-center clinical trial, patients were administered 15 mg PD-0325901 intermittently. The results exhibited a median PFS of 1.8 months and a median OS of 7.8 months. The main side effects consisted of fatigue, rash, vomiting, diarrhea, nausea, and reversible visual disturbances (Haura et al., 2010). Selumetinib (AZD6244, ARRY-142886) is also a selective MEK inhibitor. First, in a phase II, multicenter, parallel-group study, patients received 100 mg AZD6244 twice daily or 500 mg/m^2^ pemetrexed once every 3 weeks. The results showed that the median PFS was not statistically different between the two groups (<3 months). The most common adverse events included dermatitis acneiform, vomiting, diarrhea as well as nausea (Hainsworth et al., 2010). Next, in a phase II, parallel, randomized trial, 89% of patients achieved disease stabilization. The median PFS was 4 months and the median OS was 10.5 months. The common side effects were similar (Carter et al., 2016). Furthermore, trametinib (GSK1120212) is another selective inhibitor targeting MEK1/MEK2. Patients received either 2 mg trametinib orally once a day or 75 mg/m^2^ docetaxel intravenously every 3 weeks. The results demonstrated that the PFS was similar between the two groups (∼12 weeks). The most ordinary adverse events were vomiting, rash, diarrhea, nausea, and fatigue (Blumenschein et al., 2015). In conclusion, selumetinib appears to be the best among the various MEK inhibitors and leads to a longer PFS and OS.

#### Extracellular Regulated Protein Kinase Inhibitors

ERK is the culminating kinase in the MAPK pathway cascade, providing a suitable therapeutic option for KRAS-mutant cancer patients resistant to RAF or MEK inhibitors (Hatzivassiliou et al., 2012; Morris et al., 2013). The clinical development of ERK inhibitors is behind that of RAF and MEK inhibitors. Recent clinical trials have mainly focused on treating colorectal carcinoma, gastric cancer and melanoma with ERK inhibitors. The use of ERK inhibitors for the treatment of KRAS-mutant NSCLC is expected to be explored in clinical trials.

#### Phosphatidylinositol 3 Kinase Inhibitors

The PI3K/AKT/mTOR pathway is persistently activated by KRAS mutation, which contributes to cancer progression. PI3K phosphorylation attracts AKT to the plasma membrane, then inducing mTOR activation. A phase I, multicenter, dose-escalation study investigated the safety, maximum tolerated dose and preliminary activity of BKM120. The results showed that the maximum tolerated dose was 100 mg/d and this treatment was well-tolerated. Frequent side effects mainly consisted of rash, diarrhea, mood alteration, anorexia as well as hyperemia (Bendell et al., 2012).

#### Mammalian Ttarget of Rapamycin Inhibitors

mTOR is a serine/threonine kinase, activated by AKT. In a phase II, randomized, multi-center trial, 40 mg ridaforolimus was administered to patients with stage IIIB/IV KRAS-mutant NSCLC once a day. Investigators reported a median PFS of 4 months and a median OS of 18 months. The most ordinary side effects consisted of fatigue, diarrhea, mucositis, pneumonia as well as hyperglycemia (Riely et al., 2012).

#### Focal Adhesion Kinase Inhibitors

RHOA-FAK axis is a key downstream regulator in the KRAS signal transduction pathway. Defactinib is a selective ATP-competitive FAK inhibitor. In the previous phase I study, its safety was good. The Lung Cancer magazine published a phase II multi-cohort study that included patients in which other treatments had failed. Patients were divided into four cohorts: 1) KRAS mutation with cyclin dependent kinase inhibitor 2A (*CDKN2A*) negativity and tumor protein p53 (*TP53*) negativity; 2) KRAS mutation with *CDKN2A* positivity and *TP53* negativity; 3) KRAS mutation with *CDKN2A* negativity and *TP53* positivity; and 4) KRAS mutation with *CDKN2A* and *TP53* positivity). The results showed that defactinib exhibited a modest clinical activity, independent of *CDKN2A* or *TP53* status. The median PFS was 45 days. The most common side effects included fatigue, gastrointestinal problems, and increased bilirubin (Gerber et al., 2020).

To sum up, clinical trials on monotherapy for KRAS-related pathways were listed in Table 2. These inhibitors provided some but limited benefits for patients. Compared with related inhibitors, the MEK inhibitor selumetinib and mTOR inhibitor ridaforolimus exhibited a much longer PFS and OS, showing promise for further study. The common adverse events mainly included slight fatigue, gastrointestinal problem and skin problem. Due to the compensatory mechanism and complex feedback loops between the RAF/MEK/ERK and PI3K/AKT/mTOR pathways, simultaneously inhibiting multiple nodes of these pathways can lead to more sustainable and durable effects. Thus, combination treatment strategies have also been introduced below. However, it is worth noting that combination treatment may be associated with a higher toxicity.

**TABLE 2:** Completed clinical trials about therapeutic drugs targeting KRAS-related pathways in NSCLC.

Therapeutic drug	Author	Target	Study design	Patients with NSCLC	KRAS mutation	Primary endpoint	Median OS	Median PFS	Adverse effect
Sorafenib	Smit et al.	RAF	Phase I, open label, single center	10	KRAS^G12V^, KRAS^G12C^, KRAS^G12A^, KRAS^G13S^	Response	Not announced	3 months	Hand-foot syndrome (most troublesome)
Sorafenib	Dingemans et al.	RAF	Single-arm, phase II, open label, multi-center	57	KRAS codon 12, 13, or 61 mutations	Disease control rate at 6 weeks	5.3 months	2.3 months	Hand–foot reaction, cough, dyspnea, diarrhea, fatigue, anorexia
Sorafenib	Blumenschein et al.	RAF	Phase II, open label, multi-center	105	KRAS exons 1, codons 12 and 13; and exon 2, codon 61	Disease control rate at 8 weeks	8.48 months	2.83 months	Hand–foot syndrome, fatigue, rash, diarrhea, weight loss
Sorafenib	Kim et al.	RAF	Randomize, phase II, single-center, open-label	255	KRAS codon 12, 13 or 61 mutations	Disease control rate at 8 weeks	8.8 months	1.9 months	Rash, diarrhea, pain, fatigue, hand–foot syndrome…
Sorafenib	Paz-Ares et al.	RAF	Phase III, randomized, double-blind, multi-center	703	Mutant; wild-type	Overall survival	KRAS mutant vs. KRAS wild-type = 6.4 vs.5.1 months; *p* = NS	KRAS mutant vs. KRAS wild-type = 2.6 vs.1.7 months; *p* = 0.007	Skin toxicities, fatigue, diarrhea
Sorafenib	Papadimitrakopoulou et al.	RAF	Phase II, randomized, open label, multi-center	200	Mutant; wild-type	Disease control rate at 8 weeks	KRAS mutant vs. KRAS wild-type: *p* = NS	KRAS mutant vs. KRAS wild-type: *p* = NS	Fatigue (most common grade 3 to 4 toxicity)
RO5126766	Martinez-Garcia et al.	MEK/RAF	Phase I, open label, multi-center, dose-escalation	3	Not announced	Safety	Not announced	Not announced	Rash, elevation of creatine phosphokinase, diarrhea, blurred vision
RO5126766	Honda et al.	MEK/RAF	Phase I, non-randomized, open label, single-center	3	Not announced	Safety	Not announced	Not announced	Acneiform dermatitis, creatine phosphokinase elevation, ocular disorders
RO5126766	Chenard-Poirier et al.	MEK/RAF	Phase I, non-randomized, open label, single-center	10	Mutant	Response	Not announced	Not announced	Not announced
PD-0325901	Haura et al.	MEK	Phase II, open-label, multi-center	34	Not announced	Response	7.8 months	1.8 months	Fatigue, rash, vomiting, diarrhea, nausea, reversible visual disturbances
Selumetinib	Hainsworth et al.	MEK	Phase II, multi-center, open-label, randomized, two-arm	84	Not announced	Disease progression event count	Not announced	67 vs. 90 days, *p* = 0.79	Dermatitis acneiform, vomiting, diarrhea, nausea
Selumetinib	Carter et al.	MEK	Phase II, parallel, multi-center, open-label, randomized	11	Mutant	Progression free survival, Response	10.5 months	4 months	Diarrhea, rash, edema, nausea, creatine phosphokinase elevation
Trametinib	Blumenschein et al.	MEK	Phase II, multi-center, open-label, randomized	129	Mutant	Progression free survival	8 months	12 weeks	Vomiting, rash, diarrhea, nausea, fatigue
BKM120	Bendell et al.	PI3K	Phase I, multicenter, open-label, single-agent, dose-escalation	2	Mutant	Dose-limiting toxicities	Not announced	Not announced	Rash, diarrhea, mood alteration, anorexia, hyperemia
Ridaforolimus	Riely et al.	mTOR	Phase II, multi-center, open-label, randomized	79	Mutant	Progression free survival	18 months	4 months	Fatigue, diarrhea, mucositis, pneumonia, hyperglycemia
Defactinib	Gerber et al.	FAK	Phase II, single-center, open-label, multi-cohort	55	KRAS mutation at codon 12, 13, or 61	Progression free survival at 12 weeks	Not announced	45 days	Fatigue, gastrointestinal, increased bilirubin

RAF, rapidly accelerated fibrosarcoma; MEK, MAPK/ERK, kinase; PI3K, phosphatidylinositol 3 kinase; mTOR, mammalian target of rapamycin; FAK, focal adhesion kinase.

## Immunotherapy Against Kirsten Rat Sarcoma Viral Oncogene Homolog-Mutant Non-Small Cell Lung Cancer

The emergence of immunotherapy as a form of cancer treatment is a great breakthrough in the treatment of cancer (Li et al., 2019). A recent study found that in NSCLC, KRAS mutations were closely associated with confirmed biomarkers of immunotherapy, including the tumor mutation burden, programmed death-ligand 1 (PD-L1) and tumor-infiltrating lymphocytes. Patients with KRAS-mutant NSCLC may benefit from immunotherapy (Liu et al., 2020). This section primarily focuses on the use of immune checkpoint inhibitors, as well as certain immune-related cancer vaccines and adoptive cell therapy for the treatment of NSCLC.

### Use of Immune Checkpoint Inhibitors for the Treatment of Kirsten Rat Sarcoma Viral Oncogene Homolog-Mutant Non-Small Cell Lung Cancer

Anti-programmed cell death protein 1 (anti-PD-1) inhibitor, anti-programmed cell death-ligand protein 1 (anti-PD-L1) inhibitor and anti-cytotoxic T-lymphocyte associated protein 4 (anti-CTLA-4) inhibitor are common immune checkpoint inhibitors. In a randomized, open-label, phase III trial CheckMate057 (NCT01673867), patients were administered either an anti-PD-1 antibody (nivolumab; 3 mg/kg every 2 weeks) or docetaxel monotherapy (75 mg/m^2^ every 3 weeks). The results indicated that patients receiving nivolumab monotherapy had a better OS (12.2 vs. 9.4 months), while they experienced minor adverse events, including fatigue, nausea, decreased appetite, and asthenia (Borghaei et al., 2015). Moreover, in a randomized, open-label, multi-center, phase III trial OAK (NCT02008227), patients were administered either an anti-PD-L1 antibody (atezolizumab, 1,200 mg every 3 weeks intravenously) or docetaxel (75 mg/m^2^ every 3 weeks intravenously), respectively. Atezolizumab monotherapy also led to a longer median OS (17.2 vs. 10.5 months). Adverse events following atezolizumab monotherapy were fewer than following docetaxel monotherapy (Rittmeyer et al., 2017). Thus, anti-PD-1/L1 antibodies are effective in treating KRAS-mutant NSCLC. In addition, there are ongoing studies on anti-CTLA-4 inhibitors (NCT02477826 and NCT03215706) (Hellmann et al., 2019; Paz-Ares et al., 2021). Studies have also suggested that combination treatment between covalent KRAS inhibitors and immune checkpoint inhibitors is worthy of investigation (Canon et al., 2019; Hallin et al., 2020). Of note, a retrospective study reported that a KRAS^G12D^ mutation was a negative prognostic factor while a KRAS^G12C^ mutation was a positive prognostic factor following immunotherapy (Aredo et al., 2019). Besides, since high expressions of PD-L1, CD8^+^ T cells, and TMB are associated with KRAS/P53 mutations, patients with such simultaneous mutations can benefit the most from PD-1 inhibitors (Dong et al., 2017). By contrast, LKB1-loss is associated with a low PD-L1 expression, as well as a lack of infiltrating CD8^+^ TILs, resulting in innate resistance to PD-1 inhibitors of patients with KRAS-mutant NSCLC (Skoulidis et al., 2015; Listì et al., 2018). Therefore, individualized immunotherapy will be needed in the future, due to the heterogeneity of KRAS mutations.

### Use of Immune-Related Emerging Therapeutics for the Treatment of Kirsten Rat Sarcoma Viral Oncogene Homolog-Mutant Non-Small Cell Lung Cancer

In addition to common immunotherapy, certain immune-related emerging therapeutics, such as cancer vaccines and adoptive cell therapy, are being investigated in clinical studies.

The role of cancer vaccines is to motivate the immune system to recognize different antigen markers mainly expressed on tumor cells and to lyse these tumor cells (Even-Desrumeaux et al., 2011). Nowadays, various types of cancer vaccines exist, including in microparticle, nanoparticle and liposome formulations (Neek et al., 2019). By the end of 2017, Moderna had launched the candidate vaccine mRNA-5671/V941, encoding the G12C, G12D, G12V, and G13D KRAS-mutant antigens. Following an intramuscular injection, the mRNA nanoparticles are taken in by antigen-presenting cells, translated and presented on the cell surface, thereby causing T cells to respond to the new epitope of KRAS (Moore et al., 2020). A phase I, open-label, non-randomized, multi-center clinical trial on mRNA-5671/V941 as a monotherapy or combined with pembrolizumab is ongoing (NCT03948763). This study is due to be completed in 2025. However, Merck recently announced that it has abandoned its collaboration with Moderna to develop a cancer mRNA vaccine targeting KRAS mutation. The phase I clinical trial about mRNA-5671/V941 has also been discontinued. Merck noted that future partnerships with Moderna remain focused on oncology, with the two companies continuing to work together to develop personalized cancer vaccines such as mRNA-4157 as well as cancer vaccines that encode the four most common KRAS mutations. In addition to mRNA vaccines, cancer vaccines also include polypeptide vaccines and dendritic cell–based vaccines. The polypeptide vaccine is a kind of vaccine prepared by chemical synthesis technology, according to the amino acid sequence of some known or predicted epitope of the pathogen antigen gene. If the specific antigen in tumor cells can be identified and the vaccine can be developed based on the amino acid sequence, the relevant immune cells can be activated to kill tumor cells with the same specific antigen. At present, the polypeptide vaccine against KRAS-mutant NSCLC is still in the preclinical stage. The dendritic cell–based vaccine induces the generation of dendritic cells (DCs) by using the patient’s autologous monocytes *in vitro*, and then loads the corresponding tumor antigens to make DCs loaded with tumor antigens. Next, these DCs are injected into the body to stimulate the proliferation of tumor-killing lymphocytes in the body. These DCs play important roles in long-term tumor surveillance and tumor-killing to achieve the purpose of eliminating tumors. Dendritic cell-based vaccines have actually made a number of major breakthroughs in animal studies and early clinical trials, including treating KRAS-mutant pancreatic cancer, colorectal cancer and triple negative breast cancer (NCT03329248, NCT03387098, NCT03586869, NCT03136406, NCT03563157, NCT03387085). However, few clinical trials about dendritic cell-based vaccines against KRAS-mutant NSCLC has been conducted. Thus, only by finding out specific antigens in different patients with different tumors and developing relevant vaccines, can we activate the immune system of patients with specific targets and inhibit the growth of tumor cells.

Another strategy being studied is the injection of autologous T cells, which transduce the mutated KRAS-specific T cell receptors into the patients. This method is known as adoptive cell therapy (Salgia et al., 2021). Changhai Hospital is conducting a clinical trial on the activity and safety of mutant KRAS^G12V^-specific T cell receptor transduced T cell therapy for patients with pancreatic cancer. The mechanism of adoptive cell therapy is the targeting of human leukocyte antigen-matched mutant KRAS tumor cells instead of normal cells. The primary outcome measures are side effects and ORR. This study is estimated to be finished in 2023 (NCT04146298). This novel strategy has also been tested for the treatment of gastrointestinal, gastric, colon, and rectal cancers (NCT03745326). Treating KRAS-mutant NSCLC using this method should be explored in the future.

## Use of Naturally Extracted Compounds for the Treatment of Kirsten Rat Sarcoma Viral Oncogene Homolog-Mutant Non-Small Cell Lung Cancer

**FIGURE 2:**
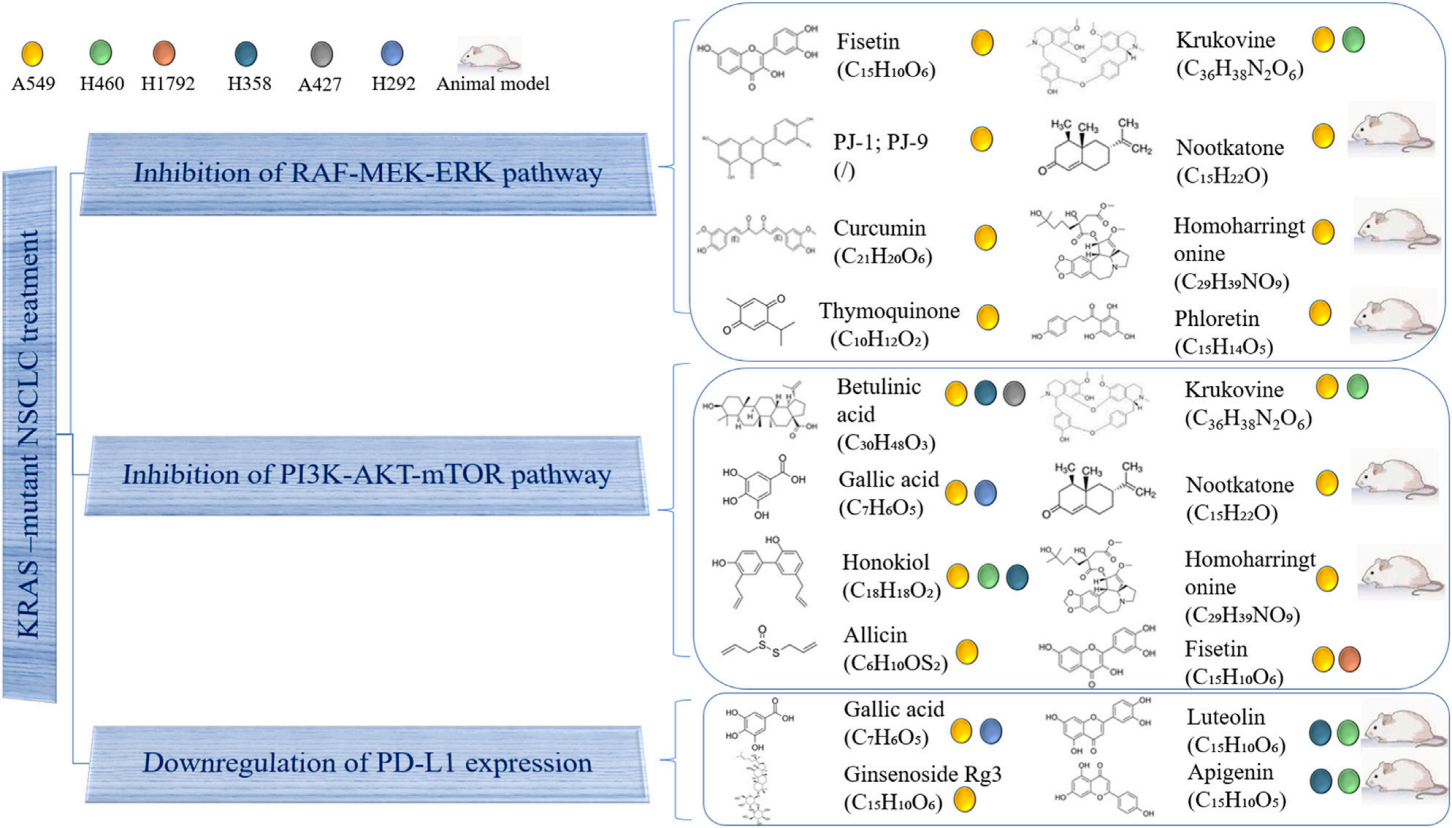
Use of natural compound treatment of KRAS-mutant NSCLC. The structure and chemical formula of natural compounds are clearly presented. The cell lines on which natural compounds have an effect are also displayed.

Although some targeted drugs have been entered into clinical studies, the underwhelming results make researchers gradually turn their attention to traditional Chinese medicines (TCMs) and their natural extracts, due to advantages such as their multi-targeted effects and low number of side effects. A previous study showed that phytochemicals were able to inhibit the proliferation of KRAS-mutated NSCLC cells in various ways, including by suppressing KRAS activation, blocking KRAS-plasma membrane interaction, inhibiting KRAS-related signaling pathways, regulating the cell cycle, promoting cell apoptosis and downregulating PD-L1 (Niloy et al., 2021). This section provides a summary of natural compounds that can battle NSCLC by suppressing the MAPK and PI3K/AKT/mTOR signaling pathways, as well as by decreasing PD-L1 expression. Table 3 and Figure 2 contain details of their mechanisms in treating KRAS-mutant NSCLC.

**TABLE 3:** Mechanisms of natural compounds in treating KRAS-mutant NSCLC.

Natural compound	Source	IC_50_/CC_50_ value	Animal type	Mechanism	References
Fisetin	Onions, cucumbers, apples, persimmons, strawberries	Not announced	Not announced	Suppress the ERK1/2 and the binding abilities of NF-κB and AP-1. Reduce the expression levels of NF-κB, c-Fos, c-Jun, MMP-2 and u-PA.	Liao et al. (2009)
PJ-1; PJ-9	*Pulicaria jaubertii*	PJ-1: CC_50_ = 1.5 mg/ml, PJ-9: CC_50_ = 1.3 mg/ml	Not announced	Reduce the expression of mutant K-Ras/B-Raf proteins, TGF-β and IL-8. Rescue p53 expression	Abd El Maksoud et al. (2019)
Curcumin	*Curcuma longa*	Not announced	Not announced	Block MEKK and ERK signaling pathways. Decrease the MMP-2 and MMP-9 expression to induce apoptosis	Lin et al. (2009)
Thymoquinone	*Nigella sativa* Linn seed oil	Not announced	Not announced	Block ERK1/2 signaling pathway to downregulate MMP-2 and MMP-9 expression. Inhibit the cell cycle	Yang et al. (2015)
Krukovine	*Abuta grandifolia*	A549: IC_50_ = 8.40 ± 0.37 μM H460: IC_50_ = 9.80 ± 0.13 μM	Not announced	Inactivate RAF-ERK pathway and AKT pathway. Induce cell cycle arrest at G1 Phase and induce apoptosis	Lai et al. (2018)
Nootkatone	Grapefruit; *Alpiniae Oxyphyllae* fructus	A549: IC_50_ = ∼ 200 µM	Athymic BALB/c male nude mice	Activate AMPK pathway, induce G1 cell arrest as well as inhibit the activation of AKT and ERK proteins	Le et al. (2019)
Homoharringtonine	*Cephalotaxus harringtonia*	Not announced	Kras^G12D^-expressing LL2 tumor-bearing mice; Kras^G12C^-driven spontaneously transgenic mice	Downregulate IL-12 expression and upregulate CD80, CD86, and CD69 expression in B220 + B cells. Reduce the oncogenic KRAS, ERK, AKT along with STAT3 protein expression	Weng et al. (2018)
Phloretin	Apple; Rosaceae plants	Not announced	Female nude mice	Decrease Bcl-2 and increase degraded form of PARP, cleaved caspase-3, cleaved caspase-9 along with BAX. Upregulate ERK1/2, JNK1/2 and P38 MAPK phosphorylation	Min et al. (2015)
Fisetin	Strawberry, apple, cucumber	Not announced	Not announced	Block PI3K-AKT-mTOR signaling	Khan et al. (2012)
Betulinic acid	*Zizyphus mauritiana*	Not announced	Not announced	Downregulate phosphorylation of AKT and mTOR. Decrease Bcl-2 and Bcl-X_L_. Enhance Bak and Bax. Induce CHOP overexpression. Activate the caspases and cleavage of PARP.	Kutkowska et al. (2017)
Gallic acid	Plants; fruits; green tea	A549: IC_50_ = 400 μM H292: IC_50_ = 100 μM	Not announced	Suppress EGFR phosphorylation. Inhibit the phosphorylation of PI3K and AKT. Activate tumor suppressor p53 and decrease PD-L1	Kang et al. (2020)
Honokiol	Magnolia tree	A549: IC_50_ = 50.58 ± 4.93 μM H460: IC_50_ = 30.42 ± 8.47 μM H358: IC_50_ = 59.38 ± 6.75 μM	Not announced	Induce apoptosis, G1 arrest, and autophagy by interrupting AMPK-mTOR signaling pathway and Sirt3/Hif-1α pathway	Luo et al. (2017)
Allicin	*Allium sativum*	Not announced	Not announced	Change TIMP/MMP balance. Inhibit PI3K/AKT pathway	Huang et al. (2017)
Luteolin/Apigenin	Vegetables and fruits	Not announced	Nude mice; xenograft mice; genetically engineered KRASLA2 mice	Induce cell apoptosis. Downregulate the IFN-γ-induced PD-L1 expression *via* inhibiting STAT3 phosphorylation	Jiang et al. (2021)
Ginsenoside Rg3	*Panax ginseng*	Not announced	Not announced	Downregulate PD-L1 expression associated with inhibition of NF-κB pathway	Jiang et al. (2017)
Tricin	*Rhizoma Phragmitis;* rice; wheat	H358: IC_50_ = 30.78 ± 1.21 μM H2122: IC_50_ = 38.46 ± 1.12 μM	Not announced	Not announced yet.	Li JX. et al. (2021)

IC_50_, inhibitory concentration 50%; CC_50_, concentration cytotoxicity 50%; ERK, extracellular signal-regulated kinase; NF-κB, nuclear factor ƙB; MMP, matrix metalloproteinase; TGF, transforming growth factor; IL, interleukin; STAT, signal transducer and activator of transcription; PD-L1, programmed death-ligand 1; PI3K, phosphatidylinositol 3-kinase; mTOR, mammalian target of rapamycin; TIMP, tissue inhibitor of metalloproteinase.

### Inhibition of MAPK Pathway

The MAPK signaling pathway can transduce extracellular signals into cells, and transmit cell signals through a tertiary kinase cascade, thereby regulating cell proliferation, differentiation, apoptosis, inflammation and exerting many other important pathological effects. The MAPK pathway has four main routes, with the RAS/RAF/MEK/ERK pathway being the most studied (Wei and Hui, 2002).

Fisetin is a natural flavonoid found in many fruits and vegetables, which has antioxidant, anti-cancer and neuroprotective effects. Dr. Liao’s team first showed that fisetin could inhibit KRAS^G12S^-mutant cancer cell A549 invasion, migration and metastasis by suppressing ERK1/2 phosphorylation, as well as the binding abilities of nuclear factor kappa B (NF-κB) and activator protein-1 (AP-1). Fisetin has also been shown to reduce the expression levels of NF-κB, c-Fos, c-Jun, matrix metalloproteinase-2 (MMP-2) and urokinase-type plasminogen activator (u-PA) (Liao et al., 2009). PJ-1 and PJ-9 are flavonoid glycosides purified from *Pulicaria jaubertii*, which inhibit A549 proliferation and metastasis by reducing the expression of mutant K-Ras/B-Raf proteins, transforming growth factor-beta (TGF-β) and interleukin 8 (IL-8). Meanwhile, the gene expression of tumor suppressor p53 was rescued (Abd El Maksoud et al., 2019). Additionally, curcumin is a natural phenolic compound extracted from *Curcuma longa* with anti-inflammatory, antioxidant, anti-proliferation and anti-angiogenesis effects. It has been demonstrated that curcumin can inhibit the invasion and migration of A549 by blocking the MEKK and ERK signaling pathways, finally inducing cell apoptosis by decreasing MMP-2 and MMP-9 expression (Lin et al., 2009). Moreover, thymoquinone, which is extracted from *Nigella sativa* Linn seed oil, has been shown to exert anti-oxidant, anti-inflammatory, anti-cancer and hepatoprotective effects. In a previous study, it blocked the ERK1/2 signaling pathway to downregulate MMP-2 and MMP-9, inhibiting the proliferation, migration and invasion of A549 cells (Yang et al., 2015). Another natural compound, krukovine, a bisbenzylisoquinoline alkaloid isolated from *Abuta grandifolia*, exhibited potent cytotoxicity to A549 and KRAS^Q12H^-mutant H460 cancer cells. It suppressed cell proliferation by inactivating the RAF-ERK and AKT pathways. It also significantly promoted cell cycle arrest at the G1 phase and induced apoptosis (Lai et al., 2018). Besides, nootkatone, derived from grapefruit and *Alpiniae Oxyphyllae* fructus, could activate the AMPK pathway, induce G1 cell arrest as well as inhibit the activation of AKT and ERK proteins. Studies have shown that either treatment with nootkatone alone or combination treatment with nootkatone and adriamycin (ADR) suppressed the progression of ADR-resistant A549/ADR lung cancer *in vivo* and *in vitro* (Le et al., 2019). Meanwhile, homoharringtonine is a cytotoxic alkaloid extracted from *Cephalotaxus harringtonia*. *In vivo*, Dr. Weng’s team used a Kras^G12D^-expressing LL2 tumor-bearing mouse model and a Kras^G12C^-driven spontaneously transgenic mouse model to demonstrate that homoharringtonine exhibited an obvious anti-cancer activity by downregulating IL-12, and upregulating CD80, CD86, and CD69 in B220 + B cells. *In vitro*, homoharringtonine suppressed A549 cell proliferation by decreasing the oncogenic KRAS, ERK, AKT, and STAT3 protein expression (Weng et al., 2018). Finally, phloretin, a polyphenolic compound found in apples and other Rosaceae plants, has been shown to induce apoptosis in A549 cells mainly by decreasing the expression of Bcl-2 and increasing that of the degraded form of PARP, cleaved caspase-3, cleaved caspase-9, and BAX. At the same time, phloretin upregulated ERK1/2, JNK1/2, and P38 MAPK phosphorylation. *In vivo*, phloretin played a significant inhibitory role in a nude mouse model (Min et al., 2015).

### Inhibition of Phosphatidylinositol 3 Kinase/AKT/Mammalian Target of Rapamycin Pathway

The PI3K/AKT/mTOR pathway is also regulated by KRAS mutations. The hyperactive PI3K/AKT/mTOR pathway reduces apoptosis and promotes the proliferation of tumor cells. Thus, certain natural compounds may exert inhibitory effects on KRAS-mutant cancer cells by blocking the PI3K/AKT/mTOR pathway.

Krukovine, nootkatone, and homoharringtonine inhibited KRAS-mutant cancer cells not only by blocking the MAPK pathway, but also by suppressing the PI3K/AKT/mTOR pathway, as discussed in the above section. In addition, fisetin could block PI3K/AKT/mTOR signaling in A549 and KRAS^G12C^-mutant NSCLC H1792 cells in a dose-dependent manner (Khan et al., 2012). Betulinic acid is a natural pentacyclic triterpenoid compound extracted from *Zizyphus mauritiana,* which has anti-inflammatory, anti-malaria, anti-AIDS and anti-tumor activities. The results showed that combination treatment with betulinic acid and sorafenib inhibited the proliferation of A549, H358 (KRAS^G12C^mutation) and A427 (KRAS^G12D^mutation) cells by downregulating AKT and mTOR phosphorylation, decreasing Bcl-2 and Bcl-X_L_ expression, increasing Bak and Bax expression, inducing CHOP overexpression and activating the caspases and cleavage of PARP (Kutkowska et al., 2017). Besides, gallic acid is a type of natural polyhydroxyphenol compound found in plant derivatives, fruits and green tea, which has antitumor, antioxidant, antibacterial, anti-inflammatory and antibacterial activities. Gallic acid has been found to suppress EGFR phosphorylation to inhibit PI3K and AKT phosphorylation. It has also been shown to activate tumor suppressor p53 and decrease the expression of PD-L1 (Kang et al., 2020). In addition, honokiol is a biphenolic phytochemical derived from the magnolia tree, which readily crosses the blood brain barrier. It obviously induced apoptosis, G1 arrest and autophagy in H460, A549, and H358 cells by interrupting AMPK/mTOR and Sirt3/Hif-1α signaling (Luo et al., 2017). Finally, allicin exhibits a high antibacterial activity and inhibits the growth of multiple microorganisms. It was considered as an anti-invasive drug through changing the tissue inhibitor of metalloproteinase (TIMP)/matrix metalloproteinase (MMP) balance and inhibiting PI3K/AKT pathway (Huang et al., 2017).

### Programmed Death-Ligand 1 Downregulation

Currently, immunotherapy is a popular cancer treatment option. In a recent preclinical study, KRAS mutations were closely associated with PD-L1 (Liu et al., 2020).

Except for gallic acid, which has previously been mentioned, luteolin and its derivative, apigenin, were found by our team to clearly suppress H358 and H460 cell proliferation, induce cell apoptosis and downregulate the IFN-γ-induced PD-L1 expression by inhibiting STAT3 phosphorylation. *In vivo*, three mouse models (nude mice, xenograft mice, and genetically engineered KRASLA2 mice) were used to test the inhibitory effect of luteolin and apigenin (Jiang et al., 2021). Additionally, ginsenoside Rg3, one of the active compounds of ginseng root, inhibits vascular endothelial growth factor expression and exhibits an antitumor activity. Dr. Jiang demonstrated that it attenuated cisplatin resistance by decreasing PD-L1 expression, which was associated with the inhibition of the NF-κB pathway in the treatment of KRAS-mutant NSCLC (Jiang et al., 2017). In addition, our research group recently found that tricin (C_17_H_14_O_7_), a flavonoid constituent isolated from *Rhizoma Phragmitis,* rice and wheat, had the ability to selectively target H358 and H2122 cells (Li JX. et al., 2021). Our preliminary results exhibited that tricin decreased PD-L1 expression in H358 and H2122 cells. The relationship between tricin and the PD-1/PD-L1 axis will be explored further by our team in the future.

In a word, naturally extracted compounds have been shown to play important roles in treating KRAS-mutant NSCLC in preclinical studies. We hope that an increasing number of compounds will enter clinical trials and benefit patients.

## Combination Strategies for the Treatment of Kirsten Rat Sarcoma Viral Oncogene Homolog-Mutant Non-Small Cell Lung Cancer

Considering the limited benefits the aforementioned monotherapies exhibited for patients, combination treatment is required to boost the anticancer effects. Currently, a large number of clinical trials are being conducted on combination treatment (Table 4).

**TABLE 4:** Combinational strategies in clinical development for treating KRAS-mutant NSCLC.

Clinical trial	Sponsor	Drug 1	Drug 2	Phase	Start date	Completion date
NCT04185883	Amgen	Sotorasib (KRAS^G12C^ inhibitor)	Afatinib (EGFR inhibitor); Pembrolizumab (anti-PD-1 inhibitor); Atezolizumab (anti-PD-L1 inhibitor)	1b/2	December 2019	January 2026
NCT03785249	Mirati Therapeutics Inc	MRTX849 (KRAS^G12C^ inhibitor)	Pembrolizumab (anti-PD-1 inhibitor); Afatinib (EGFR inhibitor)	1b	January 2019	December 2022
NCT04613596	Mirati Therapeutics Inc	MRTX849 (KRAS^G12C^ inhibitor)	Pembrolizumab (anti-PD-1 inhibitor)	2	December 2020	November 2024
NCT04449874	Genentech, Inc	GDC-6036 (KRAS^G12C^ inhibitor)	Atezolizumab (anti-PD-L1 inhibitor); Erlotinib (EGFR inhibitor)	1	July 2020	August 2023
NCT03600883	Amgen	AMG 510 (KRAS^G12C^ inhibitor)	Anti-PD-1/L1 inhibitor	1/2	August 2018	July 2026
NCT04699188	Novartis Pharmaceuticals	JDQ443 (KRAS^G12C^ inhibitor)	Spartalizumab (anti-PD-1 inhibitor)	1b/2	February 2021	August 2024
NCT03299088	University of California, Davis	Trametinib (MEK inhibitor)	Pembrolizumab (anti-PD-1 inhibitor)	1b	June 2018	May 2022
NCT02607813	Novartis Pharmaceuticals	LXH254 (RAF inhibitor)	PDR001 (anti-PD-1 inhibitor)	1	January 2016	November 2021
NCT03600701	National Cancer Institute	Cobimetinib (MEK inhibitor)	Atezolizumab (anti-PD-L1 inhibitor)	2	July 2018	July 2022
NCT01229150	National Cancer Institute	AZD6244 (MEK inhibitor)	Erlotinib (EGFR inhibitor)	2	October 2010	November 2015
NCT01392521	Bayer	Copanlisib (PI3K inhibitor)	Refametinib (MEK inhibitor)	1b	July 2011	April 2014
NCT02230553	Netherlands Cancer Institute	Lapatinib (EGFR inhibitor)	Trametinib (MEK inhibitor)	1/2	October 2014	December 2019
Clinical trial	Sponsor	Drug 1	Drug 2	Phase	Start date	Completion date
NCT01363232	Array Biopharma	BKM120 (PI3K inhibitor)	MEK162 (MEK 1/2 inhibitor)	1b	August 2011	December 2017
NCT01337765	Pfizer	BEZ235 (PI3K/mTOR inhibitor)	MEK162 (MEK 1/2 inhibitor)	1b	July 2011	March 2013
NCT01390818	EMD Serono	MSC1936369B (MEK inhibitor)	SAR245409 (PI3K/mTOR inhibitor)	1b	May 2011	April 2015
NCT03284502	Hanmi Pharmaceutical Company Limited	Cobimetinib (MEK inhibitor)	HM95573 (RAF inhibitor)	1b	May 2017	December 2023
NCT01859026	H.Lee Moffitt Cancer Center and Research Institute	MEK162 (MEK 1/2 inhibitor)	Erlotinib (EGFR inhibitor)	1/1b	December 2013	April 2023
NCT02450656	Netherlands Cancer Institute	Afatinib (EGFR inhibitor)	Selumetinib (MEK inhibitor)	1/2	June 2015	December 2019
NCT01021748	Merck Sharp and Dohme Corp	MK2206 (AKT inhibitor)	AZD6244 (MEK inhibitor)	1	November 2009	July 2014
NCT02974725	Novartis Pharmaceuticals	LXH254 (RAF inhibitor)	LTT462 (ERK inhibitor); Trametinib (MEK inhibitor)	1b	February 2017	November 2022
NCT03905148	BeiGene	Lifirafenib (RAF inhibitor)	Mirdametinib (MEK inhibitor)	1b	May 2019	April 2024

EGFR, epidermal growth factor receptor; PD-1, programmed death-1; PD-L1, programmed death-ligand 1; PI3K, phosphatidylinositol 3-kinase; mTOR, mammalian target of rapamycin; RAF, rapidly accelerated fibrosarcoma; MEK, MAPK/ERK, kinase; ERK, extracellular signal-regulated kinase; AKT, Protein Kinase B.

### Combination of Kirsten Rat Sarcoma Viral Oncogene Homolog^G12C^ Inhibitors and Anti-Programmed Cell Death Protein 1/Ligand 1 Inhibitors

One interesting combination treatment is that of KRAS^G12C^ inhibitors and anti-PD-1/L1 inhibitors. Dr. Canon found that, in a CT-26 *KRAS* ^*G12C*^ mouse model, monotherapy with covalent KRAS^G12C^ inhibitor sotorasib only caused the regression of 1/10 tumors, and so did monotherapy with anti-PD-1 inhibitor. However, combining sotorasib with anti-PD-1 antibody promoted cancer regression in 9/10 mice and extended survival (Canon et al., 2019). Thus, clinical trials evaluating the efficacy and safety of this combination treatment are ongoing, and the results are highly anticipated by patients. Considerable research attention has been paid on evaluating the combined effect of KRAS^G12C^ inhibitors such as sotorasib, adagrasib, GDC-6036 or JDQ443, and anti-PD-1/L1 inhibitors such as pembrolizumab, atezolizumab or spartalizumab (NCT04185883, NCT03785249, NCT04613596, NCT03600883, NCT04449874, and NCT04699188).

### Combination of Kirsten Rat Sarcoma Viral Oncogene Homolog^G12C^ Inhibitors and Inhibitors Targeting the Kirsten Rat Sarcoma Viral Oncogene Homolog-Related Pathways

There is also interest in the study of the combination of KRAS^G12C^ inhibitors with inhibitors targeting the KRAS-related pathways. Dr. Canon’s team conducted experiments on KRAS^G12C^-mutant cell lines, the results of which showed that combining sotorasib with EGFR, MEK, PI3K or AKT inhibitors resulted in stronger synergy. *In vivo*, sotorasib combined with the MEK inhibitor also exhibited much more obvious antitumor effects. These preclinical data suggested that this type of combination treatment might eliminate bypass signaling, which induced drug resistance (Canon et al., 2019). The combination of sotorasib (KRAS^G12C^ inhibitor) and afatinib (EGFR inhibitor) for the treatment of advanced NSCLC is being investigated (NCT04185883) in a clinical study. Another phase Ib, non-randomized, open-label trial is investigating the safety, tolerability and activity of the combination of adagrasib (KRAS^G12C^ inhibitor) and afatinib (EGFR inhibitor) (NCT03785249). Furthermore, participants with KRAS^G12C^-mutant NSCLC received GDC-6036 (KRAS^G12C^ inhibitor) in combination with erlotinib (EGFR inhibitor). This phase I dose-escalation and dose-expansion trial is still ongoing (NCT04449874; due to be completed in August 2023).

### Combination of Anti-Programmed Cell Death Protein 1/Ligand 1 Inhibitors and Inhibitors Targeting the Kirsten Rat Sarcoma Viral Oncogene Homolog-Related Pathways

Another potential approach is that of combining anti-PD-1/L1 inhibitors with inhibitors targeting the KRAS-related pathways. In a preclinical study, Dr. Liu demonstrated that MEK inhibitors combined with anti-PD-1 inhibitors increased the number of CD8^+^ tumor infiltrating lymphocytes (Liu et al., 2015). At the same time, in a model of BALB/c mice inoculated with CT26 cells, Dr. Ebert’s team found that monotherapy with anti-PD-L1 inhibitor or MEK inhibitor G-38963 only slightly inhibited tumor growth. However, combination treatment with these two inhibitors induced an obvious suppression, and in some cases led to complete regression and extended survival (Ebert et al., 2016). A phase Ib, non-randomized, open-label clinical trial is currently investigating the safety and efficacy of the combination of pembrolizumab (anti-PD-1 inhibitor) and trametinib (MEK inhibitor). The primary outcome measure is dose-limiting toxicity (NCT03299088). In addition, a phase I study on the combination of oral LXH254 (RAF inhibitor) with PDR001 (anti-PD-1 inhibitor) was recently completed, but its results have not been published as of yet (NCT02607813). Similarly, there is also a phase II clinical trial studying how effective the combination of atezolizumab (anti-PD-L1 inhibitor) and cobimetinib (MEK inhibitor) is for the treatment of patients with metastatic, recurrent and refractory NSCLC (NCT03600701; due to be completed in 2022).

### Combination Treatment With Two Different Inhibitors Targeting the Kirsten Rat Sarcoma Viral Oncogene Homolog-Related Pathways

Several preclinical studies have shown that KRAS-mutant cancer treatment required co-targeting the RAF/MEK/ERK and PI3K/AKT/mTOR pathways, due to multipoint crosstalk, negative feedback and redundancy (Carracedo and Pandolfi, 2008; She et al., 2010). The dual inhibition of these two pathways will eliminate the compensatory effect and produce an improved anti-tumor efficacy (Haagensen et al., 2012; Roberts et al., 2012). Clinically, in a phase I, non-randomized, open-label trial, patients were orally administered MEK inhibitor AZD6244 and AKT inhibitor MK2206. The results showed that the durable tumor shrinkage occurred in NSCLC and low-grade ovarian carcinoma with KRAS mutation following the combination treatment. A dose of 135 mg MK2206 weekly and 100 mg AZD6244 once a day was proposed as the recommended dose (NCT01021748) (Tolcher et al., 2015). In another phase Ib, open-label, dose-escalation, multi-center study, a lot of patients discontinued combination treatment with PI3K inhibitor BKM120 and MEK1/2 inhibitor MEK162, due to the intolerable toxicities associated with it. Despite the encouraging results in terms of efficacy, this combination strategy needs to be further explored to identify a better-tolerated dosage (NCT01363232) (Bardia et al., 2020). In addition, clinical trials on the combination of MEK and EGFR inhibitors, that of MEK and RAF inhibitors, and that of ERK and RAF inhibitors are being explored (NCT01229150, NCT02230553, NCT01337765, NCT01392521, NCT01390818, NCT03284502, NCT01859026, NCT02450656, NCT02974725, and NCT03905148).

All in all, compared with monotherapy, combination treatment may exhibit a much stronger antitumor effect. On the other hand, it may also lead to more serious adverse events. Potential approaches to overcoming toxicities include reducing the dose of both agents, evaluating the recommended dose and undertaking the pulsatile drug delivery. The advantages of pulsatile drug delivery include mitigating toxicities and delaying resistance (Bardia et al., 2020). Furthermore, natural compounds extracted from plants and herbs have fewer side effects. Therefore, combining natural compounds with western medicines may be promising.

## Conclusion, Future Challenges and Perspectives

In conclusion, further research on the treatment of KRAS-mutant NSCLC needs to be performed. New covalent KRAS^G12C^ inhibitors sotorasib and adagrasib have exhibited promising effects. The method of suppressing specific effectors of KRAS-related pathways has also provided certain benefits for patients. In addition, patients can benefit from immunotherapy with minor side effects. A few emerging therapeutics aim at the tumor immune microenvironment, such as cancer vaccines and adoptive cell therapy, are under investigation in clinical research. Moreover, certain natural compounds have been shown to inhibit KRAS-mutant NSCLC cell proliferation by inhibiting the KRAS-related signaling pathways and downregulating PD-L1. More importantly, clinical trials on combination treatment strategies are ongoing, since they have exhibited significant synergistic effects in preclinical studies. Investigating the efficacy and safety of combination treatment strategies is crucial.

Although the development of these strategies is very exciting, there are still associated challenges that need to be overcome. For example, specific inhibitors targeting other alleles, such as KRAS^G12V^, KRAS^G12D^ and KRAS^G12A^, are needed to provide personalized treatments for large patient populations. Besides, not every patient with a KRAS mutation exhibits complete response to inhibitors. Therefore, the mechanism of resistance should be fully investigated. Furthermore, NSCLC patients usually suffer from brain metastasis. Identifying inhibitors that can successfully penetrate the blood brain barrier remains a significant challenge. In addition, it is worth noting that combination treatments maybe have a higher toxicity. Thus, it is necessary for clinicians to choose the appropriate dose, dosing frequency and pulsatile dosing algorithm. On the other hand, diagnosing KRAS mutations is challenging for clinicians. If tissue samples are inadequate, liquid biopsy may be a useful tool. Liquid biopsies offer the opportunity to detect, analyze and monitor cancer in a variety of body excretions, such as blood or urine, rather than fragments of cancer tissues. This technique is easy to perform and can be sampled repeatedly (Nacchio et al., 2020). Next-generation sequencing (NGS) is a useful tool for analyzing different biomarkers in different people at the same time (Nagahashi et al., 2019). The SiRe^®^ NGS panel is a strongly analytical tool for assessing KRAS-mutant status on circulating tumor DNA in the blood (Pepe et al., 2019). Further research is required to design much more cost-effective diagnostic algorithms to coordinate the detection of clinically relevant biomarkers in tissue and blood in patients with advanced NSCLC.

**FIGURE 3:**
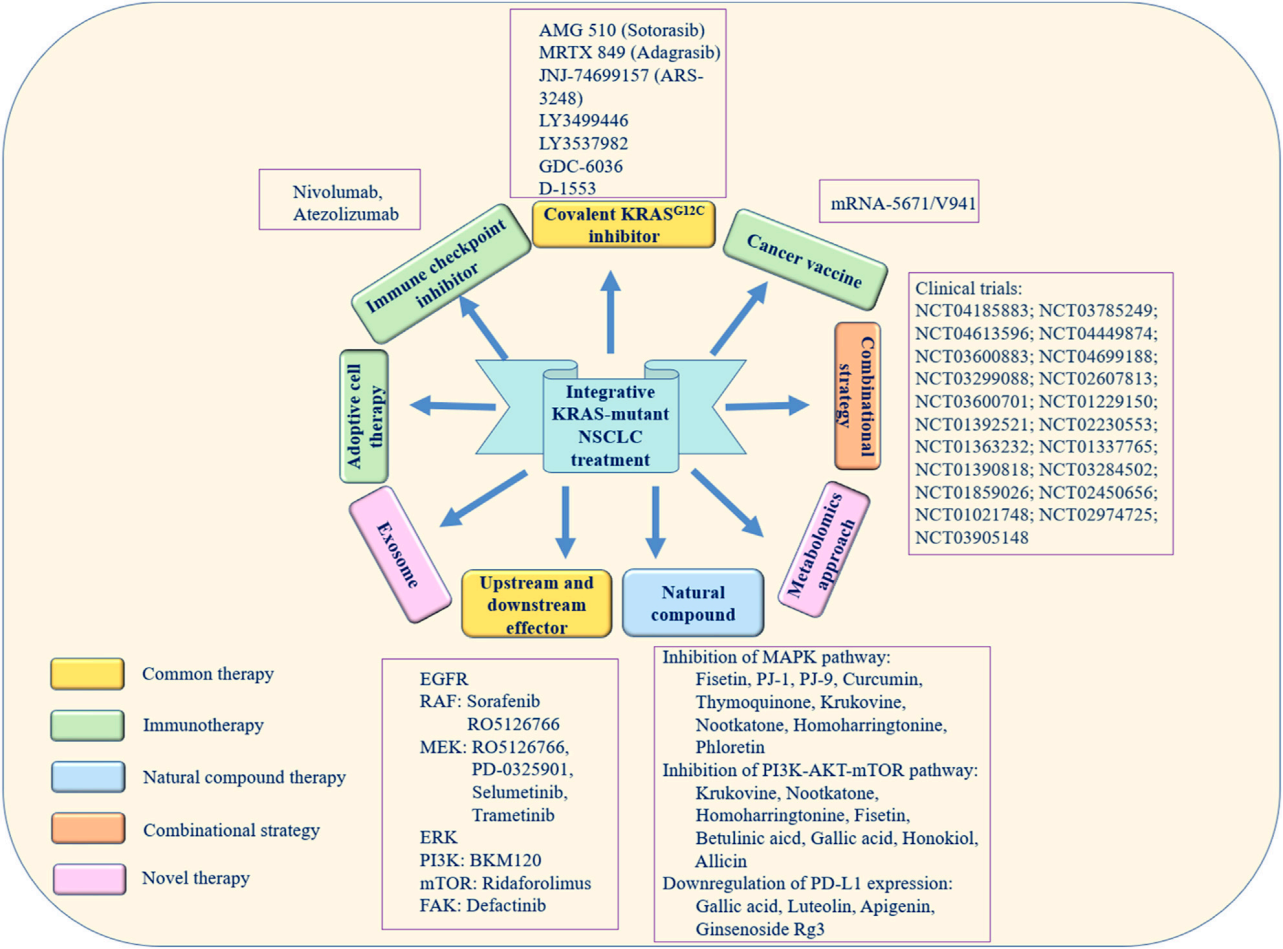
Integrative therapeutic approaches for KRAS-mutant NSCLC. The yellow boxes represent direct and indirect treatments targeting the KRAS mutation. The green boxes represent immunotherapy. The blue box represents natural compound therapy. The orange box represents combination treatment. The pink boxes represent novel therapy.

Novel methods for the treatment of cancer have recently been identified. For example, exosomes secreted by normal fibroblast-like mesenchymal cells are engineered to encapsulate the siRNA or shRNA of the KRAS^G12D^ mutation (called iExosomes). In a mouse model of KRAS^G12D^-mutant pancreatic cancer, researchers found that treatment with iExosomes could inhibit tumor growth and increase the OS of patients (Kamerkar et al., 2017). Moreover, the metabolomics approach is also a novel promising therapeutic method. In a previous study, pathway enrichment analysis was performed on the differential metabolites of KRAS homologous cell lines, and the glutathione metabolism pathway in KRAS-mutant cells was found to be abnormal. The immunohistochemistry and bioinformatics analysis results showed that SLC7A11/glutathione axis was highly expressed in KRAS-mutant lung adenocarcinoma tissues. It was demonstrated that blocking this metabolism axis could selectively inhibit the growth of KRAS-mutant lung adenocarcinoma (Hu et al., 2020). Figure 3 exhibits the aforementioned integrative therapeutics vividly.

All in all, using integrative strategies to treat KRAS-mutant NSCLC paves the way for a new branch of clinical research. Personalized precision therapy along with combination treatment may be promising options.
